# Comprehensive transcriptomic analysis integrating bulk and single-cell RNA-seq with machine learning to identify and validate mitochondrial unfolded protein response biomarkers in patients with ischemic stroke

**DOI:** 10.3389/fcell.2025.1582252

**Published:** 2025-04-17

**Authors:** Lu Zhang, Lei Yue, Peng Jia, Ziqi Cheng, Jiwen Liu

**Affiliations:** ^1^ Institute of Science and Technology for Brain-Inspired Intelligence, Fudan University, Shanghai, China; ^2^ Department of Neurology, Shangrao Municipal Hospital, Shangrao, Jiangxi, China; ^3^ Institute of Longevity and Aging Research, Zhongshan Hospital, Fudan University, Shanghai, China; ^4^ Shanghai Key Laboratory of Medical Epigenetics, Institutes of Biomedical Sciences, Fudan University, Shanghai, China; ^5^ Department of Neurosurgery, Renmin Hospital of Wuhan University, Wuhan, Hubei, China; ^6^ Department of Emergency Medicine, Shanghai Pudong New Area Gongli Hospital, Shanghai, China

**Keywords:** bioinformation, biomarker, bulk RNA-seq, ischemic stroke, single cell, mitochondrial unfolded protein response, neutrophils, virtual knockout experiments

## Abstract

**Background:**

Ischemic stroke (IS) represents a significant contributor to morbidity and mortality globally. The relationship between IS and mitochondrial unfolded protein response $\left({\text{UPR}}^{\text{mt}}\right)$ was presently uncertain. This study endeavors to explore the fundamental mechanism of ${\text{UPR}}^{\text{mt}}$ in IS by utilizing bioinformatics methods.

**Methods:**

In GSE58294, differentially expressed genes (DEGs) were obtained, which were overlapped with key module genes of ${\text{UPR}}^{\text{mt}}$ -related gene ( ${\text{UPR}}^{\text{mt}}$ -RGs) for producing candidate genes. The biomarkers were identified from the candidate genes through machine learning, expression validation, and receiver operating characteristic (ROC) curves. In order to verify the biomarkers, reverse transcription-quantitative PCR (RT-qPCR) experiments were performed on human peripheral blood. Subsequently, a predictive nomogram was created to estimate the likelihood of developing IS. Next, the mechanisms and functions related to the biomarkers were explored by enrichment analysis and immune infiltration. In addition, cells enriched with biomarkers were identified, and the biological processes involved in these cells were analyzed through intercellular communication analysis and virtual knockout experiments.

**Results:**

MCEMP1, CACNA1E, and CLEC4D were identified as biomarkers and subsequently validated by RT-qPCR. RT-qPCR revealed that CLEC4D is the most sensitive biomarker. The nomogram analysis revealed that these biomarkers possess strong diagnostic value. Immune infiltration analysis indicated that all three biomarkers are strongly correlated with neutrophils. Additionally, in the single-cell transcriptome data, these biomarkers were predominantly enriched in neutrophils. Compared to the sham group, the middle cerebral artery occlusion (MCAO) group exhibited enhanced immune-inflammatory responses. Virtual knockout experiments provide preliminary evidence that CLEC4D functions as a regulatory molecule in neutrophil-mediated inflammation, rather than serving merely as a passive marker.

**Conclusion:**

CLEC4D was identified as the most sensitive biomarker for IS related to ${\text{UPR}}^{\text{mt}}$ -RGs, offering a new reference for IS diagnosis and treatment.

## 1 Introduction

Ischemic stroke (IS) poses a significant burden on society and families due to its high incidence, mortality, and disability rates. The economic and disease burden associated with IS remains heavy (Feigin et al., 2014; Tao et al., 2020; Rochmah et al., 2021). Current treatment approaches include intravenous thrombolysis, endovascular therapy, pharmacological treatments, complication management, rehabilitation, and lifestyle modifications (Bathla et al., 2023; Maalouf et al., 2023). Although these interventions can improve outcomes for some patients, clinical treatment efficacy remains poor due to the narrow therapeutic window, potential bleeding risks, and subsequent reperfusion injury (Yang et al., 2021). Effective methods for curing brain dysfunction in IS patients are yet to be developed. Therefore, early exploration of key genes related to ischemia is crucial. Understanding the complex molecular mechanisms underlying these factors may provide new possibilities for identifying novel therapeutic targets for IS, effectively preventing severe complications and mortality associated with the disease.

During the occurrence of ischemic stroke, the interruption of blood supply impairs mitochondrial oxidative phosphorylation, leading to a sharp decline in energy production (An et al., 2021; Zhou et al., 2021; Chavda et al., 2022). Additionally, the hypoxic environment disrupts the mitochondrial electron transport chain (ETC), generating excessive reactive oxygen species (ROS) that further damage mitochondrial structure and function. In this environment, proteins within the mitochondria fail to fold correctly, or previously folded proteins undergo denaturation due to oxidative stress. When the mitochondrial protein-folding mechanisms can no longer cope with this stress, the mitochondrial stress response—known as the mitochondrial unfolded protein response $\left({\text{UPR}}^{\text{mt}}\right)$ —is activated (An et al., 2024; Jelinek et al., 2021; Chavda and Lu, 2023). ${\text{UPR}}^{\text{mt}}$ is a protective cellular response to mitochondrial dysfunction, aiming to restore mitochondrial function and prevent cell death. It regulates gene expression through various signaling pathways, promoting the repair or removal of damaged mitochondria and protecting cells from further damage by other means (Torres et al., 2024; Johns et al., 2023).

The neuroprotective role of ${\text{UPR}}^{\text{mt}}$ has been confirmed in rat models of brain injury (Sun et al., 2022). Additionally, the activation of ${\text{UPR}}^{\text{mt}}$ has been shown to promote mitochondrial protein homeostasis during adult neurogenesis and to prevent oxidative stress-related neurodegenerative diseases during aging (Zhou et al., 2022; Muñoz-Carvajal and Sanhueza, 2020). Studies also suggest that proteases associated with ${\text{UPR}}^{\text{mt}}$ play a significant role in brain inflammation following ischemia, helping to reduce the infarct area, alleviate neuroinflammation, maintain neuronal viability, and prevent mitochondrial dysfunction in the post-ischemic brain (Xiaowei et al., 2023). However, the specific molecular mechanisms of ${\text{UPR}}^{\text{mt}}$ -related genes ( ${\text{UPR}}^{\text{mt}}$ -RGs) in the pathogenesis and progression of IS remain to be further explored.

In this study, we aimed to identify potential biomarkers for IS by integrating transcriptomic data with single-cell RNA sequencing analysis. Differentially expressed genes (DEGs) from the GSE58294 dataset were cross-referenced with key ${\text{UPR}}^{\text{mt}}$ -RGs to identify candidate genes. Through machine learning and ROC curve analysis, we pinpointed MCEMP1, CACNA1E, and CLEC4D as potential biomarkers, which were then validated using reverse transcription-quantitative PCR (RT-qPCR) on human peripheral blood samples. The result of RT-qPCR shows that CLEC4D is the most sensitive biomarker. A nomogram model was developed, demonstrating the diagnostic potential of these biomarkers for predicting IS. Immune infiltration analysis revealed strong associations between these biomarkers and neutrophils, and single-cell analysis further confirmed their predominant enrichment in neutrophils. Single cellular resolution analysis revealed that the IS group showed increased immune-inflammatory responses in the peripheral blood dataset and a decline in intercellular communication in the brain cell dataset. Simulated knockout of CLEC4D uncovers its contribution to the regulation of neutrophil-induced inflammation. Our findings offer new insights into the molecular mechanisms of IS and provide valuable tools for its diagnosis and treatment.

## 2 Materials and methods

### 2.1 Data source

Ischemic stroke related transcriptomic data was obtained from the Gene Expression Omnibus (GEO) database. Specifically, GSE58294 (GPL570) includes 69 whole blood samples from IS patients (IS group) and 23 whole blood samples from healthy individuals (control group). In addition, GSE16561 (GPL6883) consists of 39 whole blood samples from IS patients (IS group) and 24 whole blood samples from healthy individuals (control group). To further validate the bulk RNA-seq results at the single-cell resolution, we utilized mouse data due to the lack of available human stroke data. Specifically, GSE225948 (GPL19057) includes 4 whole blood samples from middle cerebral artery occlusion (MCAO) mice (IS group) and 4 whole blood samples from sham surgery controls (control group). GSE154396 (GPL21273) includes 4 brain cell samples from MCAO mice (IS group) and 2 brain cell samples from sham surgery controls (control group). The description of the dataset is provided in Supplementary Table 1. In addition, 35 ${\text{UPR}}^{\text{mt}}$ -RGs were acquired from the Molecular Signatures Database (MSigDB) (Zhang et al., 2024).

### 2.2 Identification and analysis of candidate genes

Differentially expressed genes were obtained from the GSE58294 (IS vs. control) using the “limma” with P.adj<0.05 and | log fold change (FC) |>1.5 . In order to assess differences in ${\text{UPR}}^{\text{mt}}$ -RGs scores between the IS and control samples, we used the single-sample gene set enrichment analysis (ssGSEA) from the “GSVA” to analyse the ${\text{UPR}}^{\text{mt}}$ -RGs scores for all samples in GSE58294.

To further identify key genes associated with ${\text{UPR}}^{\text{mt}}$ -RG scores in IS, we performed weighted gene co-expression network analysis (WGCNA) using ${\text{UPR}}^{\text{mt}}$ -RG scores as the phenotype, on all samples from GSE58294. First, we calculated the optimal soft threshold using the “pickSoftThreshold” function to construct a scale-free network $\left(R^{2}=0.85\right)$ . After network clustering, we obtained module eigengenes (ME) from the first principal component of each clustered module and calculated the correlation between MEs and the phenotype. The module with the strongest correlation to the phenotype was selected. Key genes were then filtered based on Gene Significance (GS) and Module Membership (MM) scores. The key phenotype-related genes were intersected with DEGs to identify candidate genes.

### 2.3 Functional analysis of candidate genes

To investigate the biological functions and signaling pathways associated with the candidate genes in the pathogenesis of IS, we first performed Gene Ontology (GO) enrichment analysis using the R package “clusterProfiler.” This analysis focused on three key aspects: biological processes (BP), cellular components (CC), and molecular functions (MF), with a significance threshold of P<0.05 .

Next, to explore the potential interactions between the candidate genes at the protein level, we constructed a protein-protein interaction (PPI) network using the Search Tool for the Retrieval of Interacting Genes (STRING) database.

### 2.4 Identification of biomarkers via machine learning

We employed the Least Absolute Shrinkage and Selection Operator (LASSO) regression and Random Forest (RF) models to identify genes that significantly contribute to the accurate prediction of IS group from the identified candidate genes. Specifically, we used the candidate genes as independent variables to predict the IS group, with the weights of the candidate genes serving as training parameters. LASSO regression was applied to identify genes whose weights were not penalized to zero. In the RF model, the top eight genes ranked by importance were selected for further analysis. The intersection of the genes identified by both models was considered as the final set of candidate key genes.

Expression validation of candidate key genes was conducted in the datasets GSE58294 and GSE16561 using the Wilcoxon rank-sum test. Candidate key genes that exhibited significant differential expression (P<0.05) and consistent expression trends across both datasets were identified as hub genes.

To identify biomarkers, receiver operating characteristic (ROC) curves were generated for all hub genes. The area under the curve (AUC) values were calculated to assess the diagnostic accuracy for IS. Hub genes with an AUC > 0.7 were defined as biomarkers. In addition, we compared the AUC values of our proposed biomarkers against those of several well-established ischemic stroke biomarkers—including MMP9, MMP2, MMP3, TIMP1, APOE, APOA1, APOB, and APOC3—across two independent datasets (GSE58294 and GSE16561) (Cuadrado et al., 2009; Allard et al., 2004; Lopez et al., 2012).

### 2.5 Construction of nomogram

To facilitate patient assessment in medical research and clinical practice, we constructed a regression model using the expression levels of biomarkers to predict the probability of disease in patients. The calibration curve of the nomogram was evaluated for prediction accuracy using the “regplot” package in R. The reliability of the nomogram was assessed through decision curve analysis (DCA) using the “rmda” package. Additionally, the “pROC” package was used to generate the ROC curve for the nomogram, with the AUC serving as a measure of diagnostic accuracy for IS. An AUC value greater than 0.7 was considered indicative of good performance.

### 2.6 Gene set enrichment analysis

To investigate the biological roles associated with the biomarkers and the differences in the signaling pathways involved, we performed the GSEA analysis. We used the “stats” package in R to calculate the Spearman correlation coefficients between the biomarkers and all other genes. Subsequently, genes were ranked based on their correlation coefficients, generating a list of genes correlated with each key gene. Enrichment analysis was then performed using the GSEA function from the “clusterProfiler” package in R. The background gene set was derived from the “c2. cp.kegg.v2023.1. Hs.symbols.gmt” file in the MsigDB database.

### 2.7 Immune infiltration analysis

To further assess the differences in immune status between the IS and control groups, immune cell infiltration was analyzed using the ssGSEA algorithm from the “GSVA” package in R. This analysis generated immune scores for each of the 28 immune cell types. The Wilcoxon rank-sum test function from the “stats” package in R was used to evaluate the differences in immune cell infiltration between the IS and control groups, with differential immune cells being selected based on a significance threshold of P<0.05 . Spearman correlation analysis was then performed between the differential immune cells and biomarkers ( | cor |> 0.3, P<0.05 ).

### 2.8 Analysis of biomarker on single cell

To validate the bulk RNA sequencing results, we analyzed the expression of biomarkers at the single-cell level. We clustered the single-cell RNA sequencing (scRNA-seq) data to identify distinct cell types and examined the differences in cell type proportions and biomarker expression between the IS and control groups within each cell type. Additionally, we employed the CellPhoneDB tool to explore the intercellular communication network. To further explore the biological function of the biomarker in specific cell populations, we conducted virtual knockout (*in silico* KO) analyses. Single-cell RNA sequencing datasets derived from the peripheral blood and brain cells of stroke model mice were independently analyzed using a computational knockout approach (scTenifoldpy).

### 2.9 Participants

This study was carried out in accordance with the Declaration of Helsinki established by the World Medical Association and approved by the Institutional Review Board of Shanghai Pudong New Area Gongli Hospital (GLYYls 2024-078). The inclusion criteria were as follows: patients with no history of traumatic brain injury, brain tumors, stroke, or obvious neurological deficits within the past 3 months; no use of antiplatelet drugs, anticoagulants, or neuroprotective agents. The stroke onset in patients was within 3 days. A total of 21 stroke patients were recruited based on diagnosis by experienced neurologists and supporting imaging evidence, including 11 females and 10 males, with an average age of 68.67 years (SD = 9.92). The control group consisted of 26 participants, including 13 females and 13 males, with an average age of 66.15 years (SD = 8.70). All participants, or their legal representatives, provided written informed consent. Baseline characteristics of participants is in Table 1.

**TABLE 1:** Baseline characteristics of participants.

Variables	Total (n = 47)	Control (n = 26)	Stroke (n = 21)	Statistic	P
Age, Mean ± SD	67.28 ± 9.24	66.15 ± 8.70	68.67 ± 9.92	t = −0.92	0.360
NIHSS, M (Q_1_, Q_3_)	0.00 (0.00, 8.00)	0.00 (0.00, 0.00)	9.00 (7.00, 12.00)	Z = −6.40	< 0.001
MBI, M (Q_1_, Q_3_)	100.00 (70.00, 100.00)	100.00 (100.00, 100.00)	70.00 (50.00, 80.00)	Z = −6.40	< 0.001
Gender, n (%)				$\chi^{2}$ = 0.03	0.871
Male	23 (48.94)	13 (50.00)	10 (47.62)		
Female	24 (51.06)	13 (50.00)	11 (52.38)		
mRS, n (%)				–	< 0.001
0	26 (55.32)	26 (100.00)	0 (0.00)		
1	3 (6.38)	0 (0.00)	3 (14.29)		
2	7 (14.89)	0 (0.00)	7 (33.33)		
3	6 (12.77)	0 (0.00)	6 (28.57)		
4	5 (10.64)	0 (0.00)	5 (23.81)		

t, t-test; Z, Mann–Whitney test; $\chi^{2}$ , Chi-square test; –, Fisher exact.

SD, standard deviation; M, median, Q_1_, 1st Quartile; Q_3_, 3rd Quartile.

### 2.10 Real-time, fluorescence-based quantitative PCR

Peripheral blood samples from all participants were collected in EDTA anticoagulant tubes. GAPDH was used as the internal control gene, and the expression levels of three target genes (primer information can be found in Supplementary Table 2) were detected using SYBR GREEN I to analyze the target gene expression in the submitted samples. The primary instruments used were the analytikjena-qTOWER2.2 real-time PCR system (Germany), SCILOGEX D3024R centrifuge (United States), analytikjena-Easycycler PCR machine (Germany), scandrop100 microvolume nucleic acid and protein quantifier (Germany), pipettes (Bio-Rad, United States), and the M1 q-PCR automatic pipetting system (Sichuan Kejin). The main reagents and consumables used were the TUREscript 1st Strand cDNA Synthesis Kit (Aidelai, China), 2× SYBR^®^ Green Premix (DF, China), 10 μ L tips (GCS, United States), 200 μ L tips (GCS, United States), 1 mL tips (GCS, United States), 200 μ L RNase-free PCR reaction tubes (AXGEN), 1.5 mL RNase-free EP tubes (GCS), low-profile white PCR reaction tubes (Bio-Rad), and optical sealing films (Bio-Rad). All tips and EP tubes were sterilized and dried prior to use. Other chemicals included Trizol, chloroform, isopropanol, 75% ethanol, and ultrapure water. RT-qPCR was utilized to confirm the expression levels of the biomarkers. Extraction of total RNA was carried out from 47 samples using Trizol. The total RNA was converted into cDNA utilizing the SureScript First-strand cDNA Synthesis Kit. RT-qPCR was carried out with the analytikjena-qTOWER2.2 real-time PCR system.

### 2.11 Statistical analysis

Data analysis was performed using GraphPad Prism 10. Normality tests were conducted first. For data that followed a normal distribution, Student’s t-test was applied. Data that did not pass the normality test were analyzed using the nonparametric unpaired Mann-Whitney U test. P<0.05 was considered statistically significant.

## 3 Results

### 3.1 A total of 17 candidate genes (differential ${\text{UPR}}^{\text{mt}}$ -RGs) are identified

**FIGURE 1:**
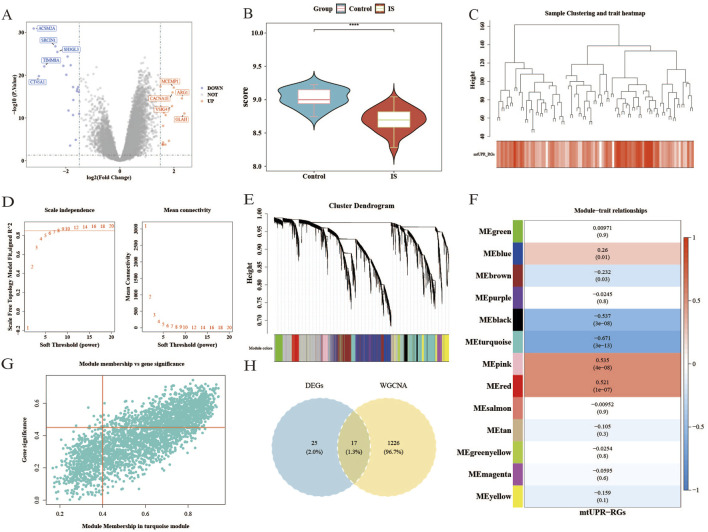
The gene expression profiles of the control group and the IS group, and weighted gene co-expression network analysis in GSE58294. **(A)** Volcano map of upregulated and downregulated genes in the IS group compared to the control group. **(B)** The differences in ssGSEA scores of ${\text{UPR}}^{\text{mt}}$ -RGs (Wilcoxon test, **** P<0.0001 ). **(C)** Hierarchical clustering and ssGSEA scores of ${\text{UPR}}^{\text{mt}}$ -RGs. **(D)** Soft threshold selection. **(E)** Co-expression module identification. **(F)** Heatmap of the correlation between gene modules and traits. **(G)** Key gene module. **(H)** Venn diagram of differential ${\text{UPR}}^{\text{mt}}$ -RGs.

In the GSE58294 dataset, we identified 42 DEGs in the IS group compared to the control group, of which 22 genes were upregulated and 20 genes were downregulated (Figure 1A). Additionally, the ${\text{UPR}}^{\text{mt}}$ -RG scores in the IS group were significantly lower than those in the control group (**** P<0.0001 ) (Figure 1B), suggesting that this score can be used for subsequent analyses. To ensure the accuracy of the following analyses, we performed clustering on the samples and removed any outliers. Figure 1C shows that no outliers were detected in this study, so all samples were retained for further analysis.

The results from the WGCNA indicated that the optimal soft threshold (β) was 8 (Figure 1D). A total of 13 modules were identified through the construction of the co-expression network (Figure 1E). Among these modules, the turquoise module, which was associated with the ${\text{UPR}}^{\text{mt}}$ -RG scores ( | cor | = 0.671, P<0.05 ), was selected as the key module, leading to the identification of 1,243 key module genes (Figures 1F,G). The intersection of the DEGs and the key module genes yielded 17 candidate genes (Figure 1H): LILRA5, ARG1, INSC, ANKRD22, MARC1, CLEC4D, BMX, CD177, MCEMP1, VSIG4, FCAR, CACNAIE, CASP5, NT5DC4, SLC26A8, GRB10, and OLAH.

### 3.2 Immune defense, apoptosis, and metabolic regulation are the primary processes associated with the candidate genes

**FIGURE 2:**
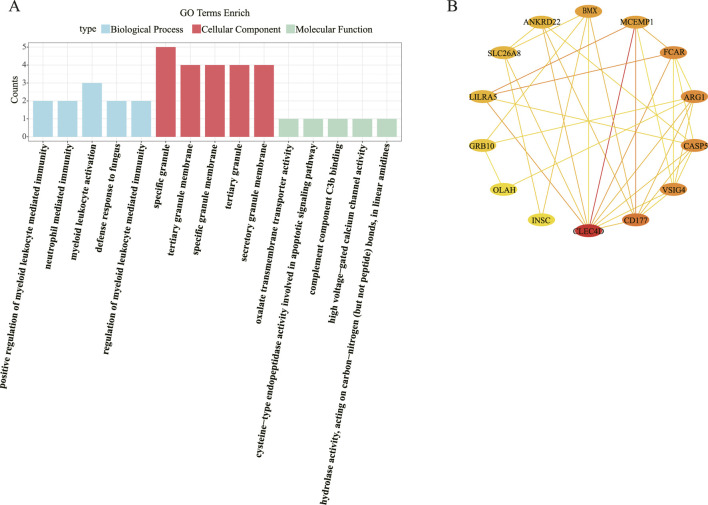
GO enrichment and PPI network analysis. **(A)** GO enrichment. **(B)** PPI network analysis.

A total of 249 GO biological functions, including 201 biological process (BP), 32 molecular function (MF), and 16 cellular component (CC) were enriched for candidate genes (Figure 2A). Functional enrichment analysis showed that in the BP, these candidate genes were primarily associated with positive regulation of myeloid leukocyte mediated immunity, neutrophil mediated immunity, myeloid leukocyte activation, defense response to fungus; In the CC, these candidate genes were mainly involved in specific granule, tertiary granule membrane, specific granule membrane, tertiary granule, secretory granule membrane; In the MF, these candidate genes were mainly involved in oxalate transmembrane transporter activity, cysteine-type endopeptidase activity involved in apoptotic signaling pathway, complement component C3b binding, high voltage-gated calcium channel activity, hydrolase activity, acting on carbon-nitrogen (but not peptide) bonds, in linear amidines.

The PPI network constructed based on candidate genes contained 35 interactions of 17 proteins. Notably, the CLEC4D and MCEMP1 interact with most of the proteins in the network (Figure 2B).

### 3.3 MCEMP1, CACNA1E and CLEC4D are identified as biomarkers

**FIGURE 3:**
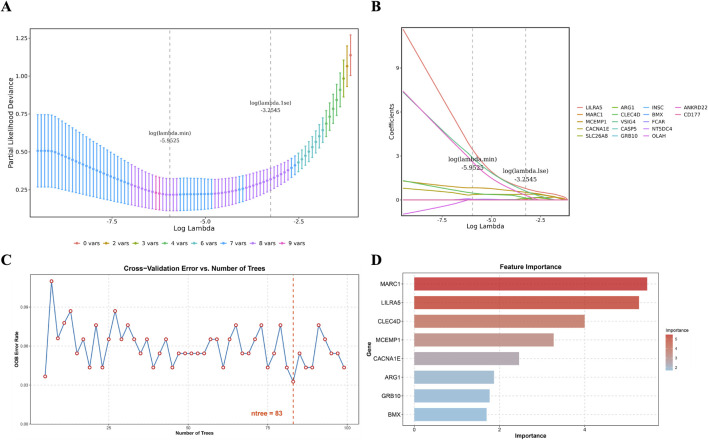
Candidate key genes were identified by machine learning. **(A)** Fitting deviation against the log(lambda) sequence. **(B)** The LASSO coefficients of the 17 candidate genes. **(C)** The relationship between the out-of-bag (OOB) error rate and the number of trees (ntree) in the random forest model. **(D)** Importance ranking of differential ${\text{UPR}}^{\text{mt}}$ -RGs (top 8).

**FIGURE 4:**
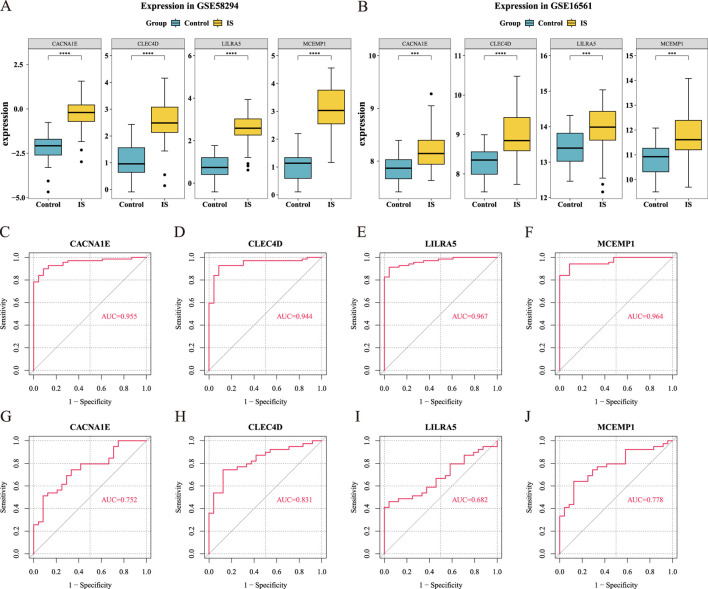
Validation of hub gene expression levels (Wilcoxon test, *** P<0.001 , **** P<0.0001 ), and ROC curve of the hub genes. **(A)** Expression in GSE58294. **(B)** Expression in GSE16561. **(C–F)** ROC curve of the hub genes in GSE58294. **(G–J)** ROC curve of the hub genes in GSE16561.

Utilizing LASSO analysis, the candidate genes with the smallest error (lambda.min = 0.0026) were selected, resulting in eight candidate characterization genes (Figures 3A,B). Additionally, Random Forest (RF) analysis (“ntree = 83”) identified another set of eight candidate characterization genes (Figures 3C,D). The intersection of these two gene sets resulted in four key candidate genes: LILRA5, MCEMP1, CACNA1E, and CLEC4D. To validate the expression levels of these four selected genes, we conducted an analysis in the GSE58294 and GSE16561 datasets. The results revealed significantly higher expression of these genes in the IS group compared to the control group, with consistent expression trends (*** P<0.001 , **** P<0.0001 ). Consequently, LILRA5, MCEMP1, CACNA1E, and CLEC4D were selected as hub genes for further investigation (Figures 4A, B).

To evaluate the diagnostic potential of these genes in distinguishing IS from control groups, we used ROC curve analysis. The results showed that the AUC values for MCEMP1, CACNA1E, and CLEC4D were greater than 0.7 in both GSE58294 and GSE16561, indicating that these three hub genes are potential biomarkers (Figures 4C–J). Comparison with well-established ischemic stroke biomarkers revealed that CLEC4D consistently achieved the highest average AUC (0.944 in GSE58294 and 0.831 in GSE16561), outperforming all classical biomarkers. Notably, MMP9 exhibited relatively high AUC values (0.863 in GSE58294 and 0.862 in GSE16561), but still slightly lower than CLEC4D (Supplementary Figure 3).

### 3.4 CLEC4D is a sensitive biomarker validated by RT-qPCR

**FIGURE 5:**
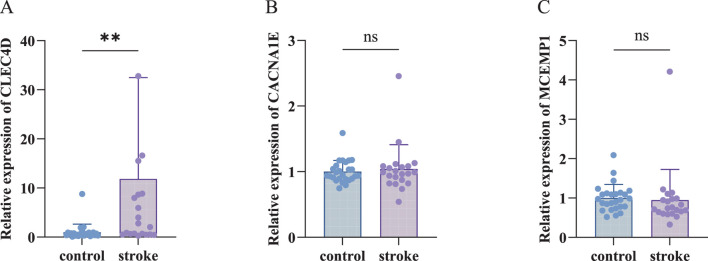
RT-qPCR Validation of biomarkers. **(A)** Relative expression of CLEC4D (nonparametric unpaired Mann-Whitney U test, ** P<0.01 ). **(B)** Relative expression of CACNA1E (nonparametric unpaired Mann-Whitney U test, ns, P>0.05 ). **(C)** Relative expression of MCEMP1 (nonparametric unpaired Mann-Whitney U test, ns, P>0.05 ).

To validate the expression levels of the identified biomarkers (CLEC4D, CACNA1E, and MCEMP1) in IS, we performed RT-qPCR analysis using peripheral blood samples from recruited participants. The RT-qPCR results (Figure 5; Supplementary Table 2) revealed that CLEC4D expression was significantly upregulated in the IS group compared to the healthy controls (** P<0.01 , Wilcoxon rank-sum test). This finding is consistent with our bioinformatics analysis, supporting the potential role of CLEC4D as a sensitive biomarker for IS. In contrast to CLEC4D, no significant differences in expression levels were observed for CACNA1E and MCEMP1 between the IS patients and the control group (ns, Wilcoxon rank-sum test). This discrepancy between the bioinformatics predictions and experimental validation may suggest that CACNA1E and MCEMP1 play less prominent roles in peripheral blood during the acute phase of IS or that their expression is regulated in a tissue-specific manner.

### 3.5 The nomogram based on biomarkers is a valuable clinical assessment scale

**FIGURE 6:**
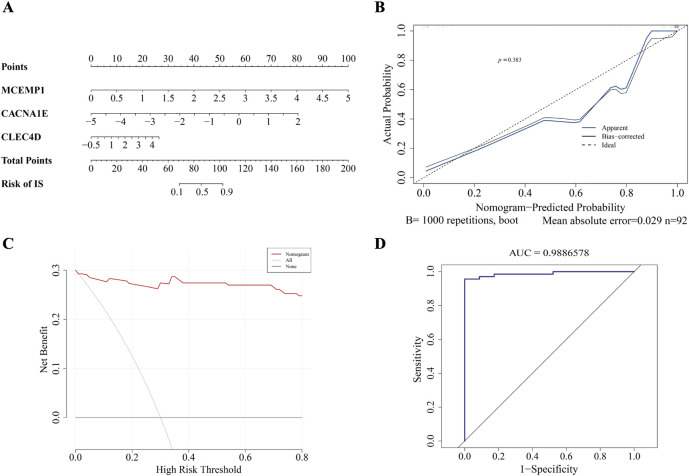
The construction of nomogram. **(A)** Nomogram. **(B)** Calibration curve. **(C)** DCA curve. **(D)** ROC curve.

In order to predict the probability of having IS by integrating biomarker, we constructed a nomogram. The nomogram assigns a point to each sample in GSE58294 based on the expression levels of the biomarkers. Each biomarker corresponds to a specific point, and the total point is obtained by summing the individual points of all biomarkers. The total point is then used to infer the likelihood of ischemic stroke (IS), with a higher point indicating an increased risk of IS. (Figure 6A). The good consistency of the calibration curve to the ideal curve indicated that the nomogram was more accurate (P=0.383) (Figure 6B). The DCA curve shows that the nomogram (red line) exhibited significantly treatment benefit compared to the “ALL” line (The benefits of treatment for all individuals) and the “None” line (The benefits of no treatment). These results showed that the nomogram demonstrated favorable clinical net benefits, suggesting its strong clinical applicability (Figure 6C). The AUC value of the ROC was 0.989, indicating that the nomogram performed well clinical utility (Figure 6D).

### 3.6 GSEA identifies CLEC4D linked to immunity and protein homeostasis pathways

**FIGURE 7:**
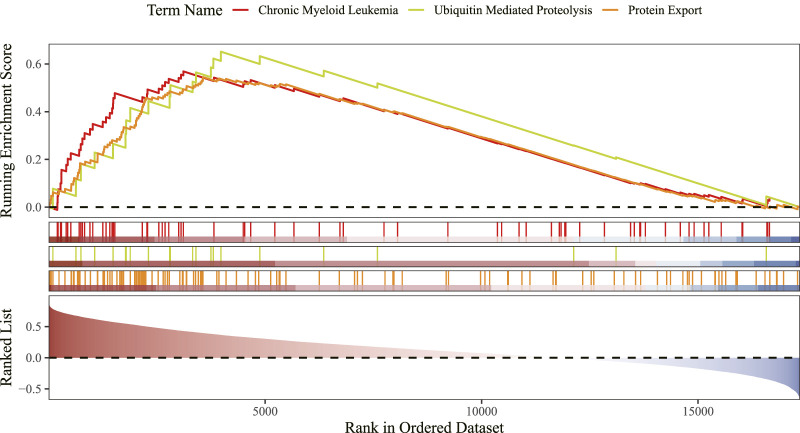
Gene set enrichment analysis (GSEA) of CLEC4D.

GSEA showed that CLEC4D was enriched in 37 functional pathways, with the top three being chronic myeloid leukemia, ubiquitin-mediated proteolysis, and protein export signaling pathway (Figure 7; Supplementary Table 3).

### 3.7 Immune infiltration analysis reveals neutrophils as the dominant biomarker carriers

**FIGURE 8:**
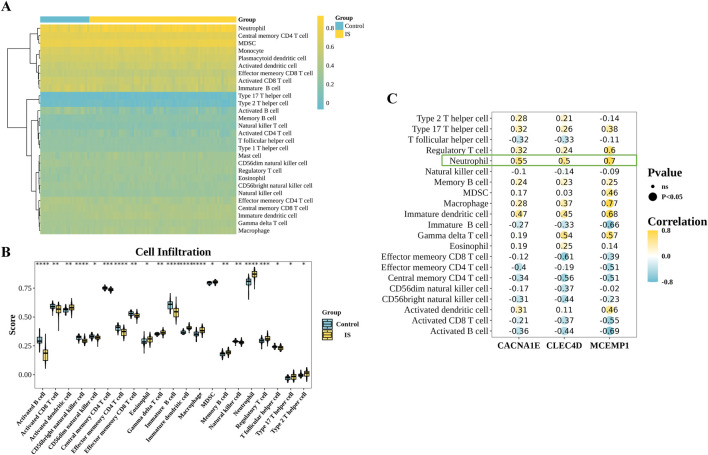
Immunoinfiltration analysis. **(A)** Immune cell abundance in the IS group and control group samples. **(B)** Differentially infiltrated immune cells between the IS group and control group samples (Wilcoxon test, **** P<0.0001 , *** P<0.001 , ** P<0.01 , * P<0.05 , ns, P>0.05 ). **(C)** A heatmap of the correlation between biomarkers and differentially infiltrated immune cells.

Immune cell infiltration analysis was performed in GSE58294 to obtain 28 immune cell infiltration scores between the IS and control groups (Figure 8A). Then, we compared the differences in immune cell infiltration between the IS and control groups, and we got 21 differential immune cells (Figure 8B). The correlation analysis between differential immune cells and biomarkers revealed a notable positive association between CACNA1E and neutrophils (cor = 0.55, P< 0.05). The most significant positive correlation was observed between MCEMP1 and macrophages (cor = 0.77, P< 0.05). Conversely, the strongest negative correlation was found between CLEC4D and effector memory CD8 T cells (cor = −0.61, P< 0.05) (Figure 8C). The total correlation of these three biomarkers with neutrophils is the strongest.

### 3.8 Single-cell analysis reveals neutrophils as key biomarker carriers

**FIGURE 9:**
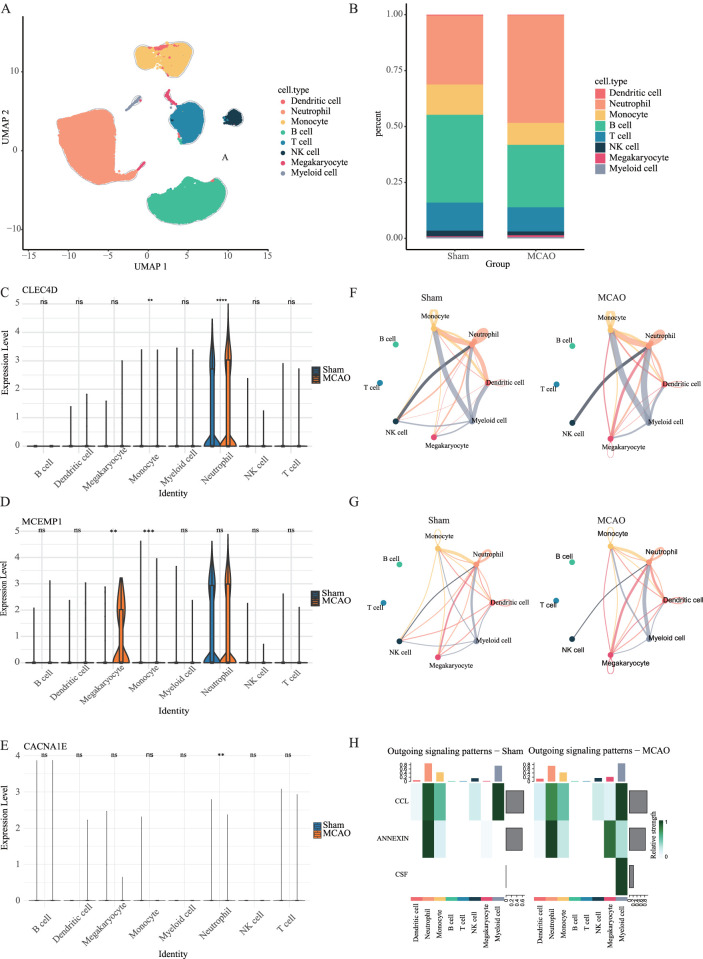
Single-cell analysis in the peripheral blood dataset. **(A)** UMAP of merged sham and MCAO groups. **(B)** The proportion of different cell types in the sham group and MCAO group. **(C–E)** The expression levels of CLEC4D, MCEMP1 and CACNA1E across different cell types in the sham group and MCAO group (ns P>0.05 , ** P<0.01 , *** P<0.001 , **** P<0.0001 ). **(F)** Interaction weights of cell-cell communication network. **(G)** Number of interactions in cell-cell communication network. **(H)** Outgoing signaling patterns in cell-cell communication analysis.

In the scRNA-seq dataset of mouse peripheral blood, we performed unsupervised clustering and uniform manifold approximation and projection (UMAP) to classify 43,269 cells into eight distinct clusters (Figure 9A). We observed a significant increase in the proportion of neutrophils in the MCAO group (Figure 9B). Furthermore, we validated the expression patterns of MCEMP1, CACNA1E and CLEC4D (Figures 9C–E) and found that these genes were predominantly enriched in neutrophils. Among them, CLEC4D exhibited significantly (**** P<0.0001 ) elevated expression in the MCAO group compared to the sham group. This result aligns with our immune infiltration analysis, further confirming the potential role of CLEC4D in neutrophil activation and inflammatory regulation after stroke.

**FIGURE 10:**
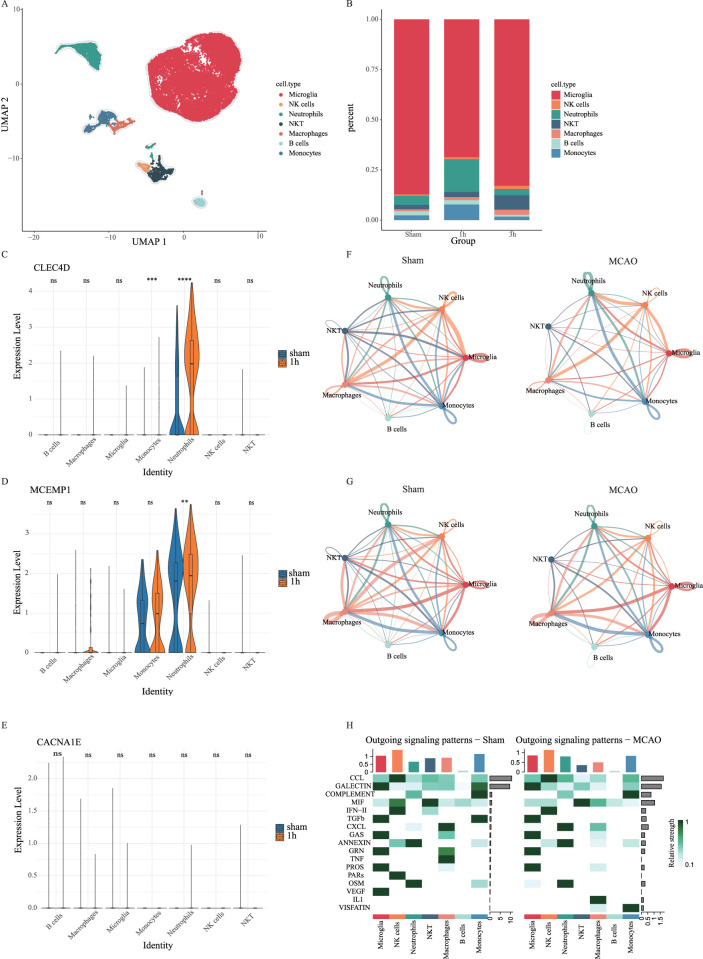
Single-cell analysis in the brain cell dataset. **(A)** UMAP of merged sham and MCAO groups. **(B)** The proportion of different cell types in the sham group, 1h-MCAO group and 3h-MCAO group. **(C–E)** The expression levels of CLEC4D, MCEMP1 and CACNA1E across different cell types in the sham group and 1h-MCAO group (ns P>0.05 , ** P<0.01 , *** P<0.001 , **** P<0.0001 ). **(F)** Interaction weights of cell-cell communication network. **(G)** Number of interactions in cell-cell communication network. **(H)** Outgoing signaling patterns in cell-cell communication analysis.

Additionally, we analyzed scRNA-seq data from brain cells. Using UMAP, we identified seven distinct cell clusters (Figure 10A). There is a significant increase of neutrophil proportion in the MCAO group within the first hour post-stroke (P<0.05) (Figure 10B). Moreover, the three biomarkers were primarily enriched in neutrophils, with CLEC4D also showing a significant (**** P<0.0001 ) upregulation (Figures 10C–E). This finding suggests that, following stroke, neutrophils not only undergo changes in peripheral blood but may also infiltrate the brain and exert crucial functions within the local microenvironment. The upregulation of CLEC4D may be closely associated with neutrophil recruitment, activation, and the exacerbation of inflammatory responses, highlighting its potential role as a key regulator of post-stroke inflammation.

### 3.9 Neutrophil-mediated cell communication is enhanced

To further investigate the role of neutrophils in the post-stroke immune microenvironment, we conducted cell-cell communication analysis using CellPhoneDB. In the peripheral blood dataset, the results revealed a significant (P<0.05) increase in interactions between neutrophils and other cell types, including monocytes, macrophages, dendritic cells, and myeloid cells, in the MCAO model (Figure 9F). Moreover, the number of communication pathways between neutrophils and myeloid cells were markedly enhanced (Figure 9G). Inflammatory signaling axes involving ANNEXIN and colony-stimulating factors (CSF) were also significantly upregulated in the MCAO group (Figure 9H). These increased intercellular communications likely facilitated neutrophil recruitment and activation, thereby amplifying the local inflammatory response.

Additionally, cell-cell communication analysis in brain tissue indicated a reduction in the number of intercellular communication pathways in the MCAO group due to cerebral infarction. However, interactions between neutrophils, macrophages, monocytes, and microglia were enhanced (Figure 10F), along with an increase in their communication pathways (Figure 10G). Compared to the sham group, the MCAO group exhibited vascular endothelial growth factor (VEGF) dysfunction in microglia, increased IL-1 signaling in macrophages, upregulated VISFATIN expression in monocytes, and enhanced CXCL signaling in neutrophils (Figure 10H). These findings suggest that neutrophils, macrophages, and monocytes may contribute to blood-brain barrier (BBB) disruption by intensifying their interactions with endothelial cells, ultimately exacerbating brain tissue damage. This study provides new insights into the neuroinflammatory response following stroke and suggests that targeting neutrophil-endothelial cell communication could be a potential therapeutic strategy for post-stroke inflammation.

### 3.10 Virtual knockout of CLEC4D reveals its regulatory role in neutrophil-mediated inflammation

**FIGURE 11:**
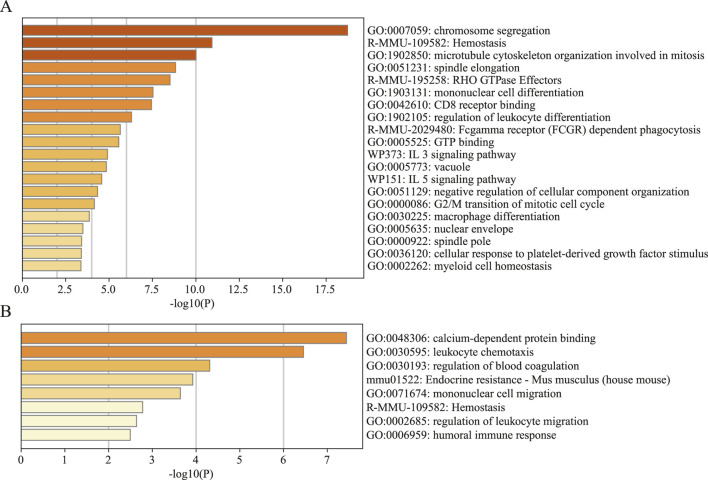
Gene set enrichment analysis (GSEA) of significantly altered genes by knocking out CLEC4D. **(A)** Brain cell sample. **(B)** Peripheral blood sample.

In brain cell-derived immune cells, virtual knockout of CLEC4D in neutrophils led to significant transcriptional reprogramming. Differential gene expression analysis followed by enrichment analysis revealed prominent involvement of pathways associated with chromosome segregation, spindle elongation, Fc γ receptor (FCGR)-dependent phagocytosis, Rho GTPase signaling, and mononuclear cell differentiation (Figure 11A; Supplementary Table 3 GSEA of CLEC4D). These findings suggest that CLEC4D is intricately involved in coordinating cytoskeletal organization and Fc receptor-mediated immune signaling in the ischemic brain microenvironment.

In contrast, the CLEC4D knockout in peripheral blood-derived cells induced a more limited response, with enrichment mainly in calcium-dependent protein binding, leukocyte chemotaxis, and regulation of blood coagulation (Figure 11B; Supplementary Table 3 GSEA of CLEC4D). This discrepancy highlights the tissue-specific functional dynamics of CLEC4D, suggesting a more active immunomodulatory role within the brain parenchyma following stroke.

Collectively, these data support the notion that CLEC4D is not merely a surface marker but likely contributes to the activation and effector functions of neutrophils in a context-dependent manner, particularly within the inflamed brain.

## 4 Discussion

We first identified key genes associated with IS and ${\text{UPR}}^{\text{mt}}$ using methods such as WGCNA and machine learning, based on bulk RNA-seq data. These key biomarkers, CLEC4D, MCEMP1, and CACNA1E, were all significantly upregulated in the IS group compared to the control group. Next, we validated CLEC4D as a relatively sensitive biomarker through RT-qPCR experiments on human peripheral blood. Finally, at the single-cell resolution, we found that these biomarkers were primarily enriched in neutrophils, with CLEC4D showing significant upregulation in the MCAO group, consistent with the bulk RNA-seq results. This finding suggests that CLEC4D may play a critical role in the inflammatory process of ischemic stroke, particularly in neutrophil-mediated inflammation.

IS leads to severe tissue damage due to the lack of blood supply, resulting in cell death and subsequent release of intracellular components. These released molecules, known as damage-associated molecular patterns (DAMPs), are crucial in triggering inflammation and immune responses. Major DAMPs involved in IS include high-mobility group box 1 (HMGB1) and peroxidases (PRX). After cell death, these molecules leak into the extracellular space and act as danger signals to the immune system (Nakamura et al., 2020; Simats and Liesz, 2022; Alsbrook et al., 2023).

DAMPs are released from ischemic cells and recognized by pattern recognition receptors (PRRs) expressed on immune cells such as microglia, macrophages, and neutrophils. For instance, HMGB1 increases the expression of matrix metalloproteinase 9 (MMP9), which contributes to blood-brain barrier (BBB) disruption and exacerbates inflammation (Li et al., 2024; Liao et al., 2024). Additionally, HMGB1 activates neutrophils and induces the release of neutrophil extracellular traps (NETs), further promoting the inflammatory cascade (Gao et al., 2024b). PRX, a protein that reduces oxidative stress by metabolizing hydrogen peroxide, is also released from ischemic cells and activates microglia and infiltrating macrophages. This activation promotes the release of pro-inflammatory cytokines such as TNF- α , IL-1 β , and IL-6, thereby enhancing immune cell recruitment, amplifying inflammation, and contributing to neuronal damage (Tan et al., 2020). DAMPs can also trigger various stress responses, including UPRmt, which plays a critical role in cellular adaptation to ischemic injury (Gao et al., 2024a).

Based on our findings, one key PRR in this process is CLEC4D. CLEC4D is a C-type lectin receptor primarily expressed on myeloid cells, including neutrophils and monocytes. CLEC4D plays an essential role in pathogen recognition, phagocytosis, and the initiation of inflammatory responses (Guerrero-Juarez et al., 2023). Our GSEA further revealed that CLEC4D is significantly enriched in pathways such as chronic myelogenous leukemia (CML), involving critical signaling pathways like JAK-STAT, MAPK, and PI3K-AKT (Singh et al., 2021). These pathways also play pivotal roles in post-stroke inflammation. For instance, STAT3 has been shown to mediate endogenous IL-6 to prevent apoptosis of neurons in the acute phase of cerebral ischemia (Yamashita et al., 2005). Activation of the PI3K/AKT signaling pathway can suppress the expression of pro-inflammatory factors induced by NF- κ B and convert M1 microglia into M2 microglia, thereby reducing inflammation (Joshi et al., 2015; Yang et al., 2019). Moreover, activation of the PI3K/AKT pathway can upregulate the expression of interleukin-1 receptor antagonist (IL-1RA), interleukin-10 (IL-10), and interferon- β (IFN- β ), while downregulating pro-inflammatory cytokines such as IL-1, TNF- α , IL-6, IL-8, and CXCL1 (He et al., 2022). Activated p38 MAPK can promote the production of inflammatory cytokines and induce the expression of adhesion molecules in endothelial cells, further damaging the blood-brain barrier and exacerbating ischemic injury (Sun and Nan, 2016). Once PRRs, especially CLEC4D, are activated by DAMPs, they initiate downstream signaling events that enhance inflammation through NF- κ B, MAPK, and PI3K-AKT pathways (Li et al., 2022). These pathways contribute to the production of pro-inflammatory cytokines and activation of immune cells. However, our study also suggests that CLEC4D signaling may have a broader role in regulating cellular stress responses, including ${\text{UPR}}^{\text{mt}}$ .

${\text{UPR}}^{\text{mt}}$ is a mitochondrial stress response mechanism that is triggered when the protein-folding capacity of the mitochondria is overwhelmed, a situation typically caused by ischemic damage. DAMPs activate PRRs, inducing oxidative stress and metabolic disturbances that impair mitochondrial function and trigger ${\text{UPR}}^{\text{mt}}$ . This response mitigates mitochondrial damage by promoting the refolding or degradation of misfolded mitochondrial proteins. After PRR signaling activation, several mechanisms regulate ${\text{UPR}}^{\text{mt}}$ to address mitochondrial stress. First, pro-inflammatory cytokines released during the immune response (such as TNF- α and IL-6) not only activate inflammatory pathways but also regulate key transcription factors of ${\text{UPR}}^{\text{mt}}$ , thereby initiating mitochondrial stress responses to restore mitochondrial protein folding and reduce damage (Hahn et al., 2014; El Jamal et al., 2016; Yang et al., 2007; Inigo and Chandra, 2022). Second, NRF1, a key transcription factor in ${\text{UPR}}^{\text{mt}}$ , regulates the expression of related genes following PRR activation, helping cells cope with mitochondrial protein-folding stress. Specifically, studies have shown that NRF1 promotes the degradation of mitochondrial proteins through the ubiquitin-proteasome system (UPS) in inflammatory macrophages, maintaining mitochondrial protein homeostasis (Yan et al., 2024). Our GSEA revealed significant enrichment of CLEC4D in the UPS pathway, suggesting that CLEC4D may regulate ${\text{UPR}}^{\text{mt}}$ through modulation of the UPS pathway, thereby maintaining mitochondrial function homeostasis.

The relationship between the inflammatory response triggered by DAMP-PRR interactions and ${\text{UPR}}^{\text{mt}}$ is complex and interdependent. While PRR activation by DAMPs induces inflammation and oxidative stress, it simultaneously triggers ${\text{UPR}}^{\text{mt}}$ to restore mitochondrial function. This dual response ensures that cells can better handle the metabolic and oxidative stresses resulting from ischemic injury. By alleviating mitochondrial dysfunction, ${\text{UPR}}^{\text{mt}}$ also helps mitigate the harmful effects of excessive inflammation. Moreover, ${\text{UPR}}^{\text{mt}}$ may influence the function of immune cells, such as neutrophils and macrophages, which are crucial for the inflammatory response. It has been proposed that ${\text{UPR}}^{\text{mt}}$ activation in these cells can shift their phenotype from pro-inflammatory to anti-inflammatory, helping resolve post-ischemic inflammation and promoting tissue repair (Pellegrino et al., 2014). This balance is crucial for limiting neuronal damage and promoting recovery.

However, it is important to acknowledge several limitations in this study. First, the limited sample size and sources may affect the statistical significance and biological generalizability of the data. Future research should validate these findings through multi-center, longitudinal studies. Second, we were unable to perform protein-level validation of CLEC4D expression (e.g., Western blot) due to sample and technical limitations. However, relevant evidence from the literature provides indirect support for our findings. CLEC4E, a closely related C-type lectin family member, has been shown to be significantly upregulated at both the mRNA and protein levels in microglia following ischemic stroke, as demonstrated in a recent study by Liu et al. (2023). CLEC4E (encoding the Mincle receptor) mediates proinflammatory responses by binding to damage-associated molecular patterns (DAMPs), such as properdin, and subsequently activating downstream Syk/NF- κ B/C/EBP β signaling pathways. Given the structural and functional similarities between CLEC4E and CLEC4D—both of which are involved in innate immune responses and neuroinflammatory signaling—it is plausible that CLEC4D may share similar regulatory patterns under ischemic conditions. These observations support the potential relevance of CLEC4D as a biomarker and warrant further investigation at the protein level in future studies. Moreover, we utilized two independent publicly available transcriptomic datasets (GSE58294 and GSE16561) to validate the differential expression and diagnostic performance of the candidate biomarkers, further supporting the robustness and reproducibility of our findings. Thirdly, the specific molecular mechanisms by which CLEC4D regulates ${\text{UPR}}^{\text{mt}}$ remain primarily based on correlation analyses, lacking direct functional validation. Future studies need to employ CRISPR/Cas9 gene knockout, RNA interference, and overexpression models to systematically explore the causal relationships between CLEC4D activation, downstream signaling networks, and the regulation of mitochondrial protein homeostasis. Furthermore, monitoring the dynamic changes of CLEC4D and its downstream signaling pathways at different time points post-ischemia will help clarify its spatiotemporal-specific functions in acute inflammatory responses and repair processes.

From a clinical translation perspective, CLEC4D and its associated signaling pathways represent promising therapeutic targets for ischemic stroke. As a myeloid C-type lectin receptor, CLEC4D interacts with Fc γ R and Mincle to initiate pro-inflammatory signaling in response to endogenous or microbial stimuli. Our virtual knockout analyses revealed that deletion of CLEC4D in brain-infiltrating immune cells alters gene expression programs related to Fc receptor-mediated phagocytosis, cytoskeletal organization, and mononuclear cell differentiation—pathways intimately linked to neuroinflammation and tissue remodeling. These findings suggest that modulation of CLEC4D activity could attenuate excessive neutrophil-driven inflammation in the ischemic brain without completely suppressing host defense mechanisms. Notably, similar strategies targeting the PD-L1–CLEC7A axis have been shown to restore neutrophil mobilization and reduce susceptibility to fungal infection (Yu et al., 2022), highlighting the feasibility of targeting CLR-related pathways pharmacologically. Although CLEC4D is enriched in neutrophil subsets exhibiting ${\text{UPR}}^{\text{mt}}$ -associated signatures, our results suggest that its immunomodulatory role is not confined to mitochondrial stress responses, but instead reflects a broader capacity to orchestrate innate immune activation. Therefore, therapeutic strategies aimed at fine-tuning CLEC4D expression or signaling may offer a dual benefit—mitigating localized inflammation while supporting mitochondrial and cellular homeostasis—potentially improving neuronal survival and patient outcomes. Future pharmacological studies and clinical trials will be essential to assess the safety, efficacy, and context-specific benefits of such interventions.

## 5 Conclusion

In summary, this study not only identifies the abnormal expression of key genes such as CLEC4D in ischemic stroke through large-scale data mining but also proposes a hypothesis that CLEC4D may regulate the balance between inflammation activation and ${\text{UPR}}^{\text{mt}}$ through multiple signaling pathways. These findings provide new insights into the complex interactions between post-stroke inflammation and cellular stress, laying the theoretical foundation for the development of targeted therapeutic strategies. Future research will further integrate multi-dimensional data and functional experiments to fully uncover the underlying mechanisms and clinical applications of this regulatory network.

## Data Availability

The original contributions presented in the study are included in the article/Supplementary Material, further inquiries can be directed to the corresponding authors.
