# Supplementary material for: Comprehensive transcriptomic analysis integrating bulk and single-cell RNA-seq with machine learning to identify and validate mitochondrial unfolded protein response biomarkers in patients with ischemic stroke
Source: Front Cell Dev Biol. 2025 Apr 17;13:1582252. doi: 10.3389/fcell.2025.1582252 (PMC12043589; doi:10.3389/fcell.2025.1582252)
Supplement: Supplementary file 3 [file DataSheet1.pdf]

Supplementary Material

1 SUPPLEMENTARY TABLES

Supplementary table 1: The description of the GEO dataset;

Supplementary table 2: Primer sequences and results of RT-qPCR;

Supplementary table 3: GSEA of CLEC4D.

2 SUPPLEMENTARY FIGURES

The single-cell RNA sequencing (scRNA-seq) dataset GSE225948, derived from murine peripheral blood mononuclear cells (PBMCs), was evaluated for quality control (QC) metrics to assess transcriptome coverage and sequencing depth. The dataset exhibits a mean of 403 unique genes per cell (SD = 192, Figure S1A) indicating a relatively low gene detection rate, which may suggest limited transcript diversity or insufficient sequencing depth. The total unique transcript count per cell averages 647 UMIs (SD = 412, Figure S1B), further highlighting potential challenges in capturing a comprehensive cellular transcriptome. The low transcriptomic complexity observed in this dataset could be attributed to technical limitations, such as suboptimal sequencing depth from Illumina NextSeq 500, or biological factors, such as the inherently lower RNA content in PBMCs. Given these QC metrics, additional data normalization and filtering steps may be necessary to enhance downstream analyses, although the dataset still provides a foundational basis for broad immune cell profiling and differential gene expression studies.

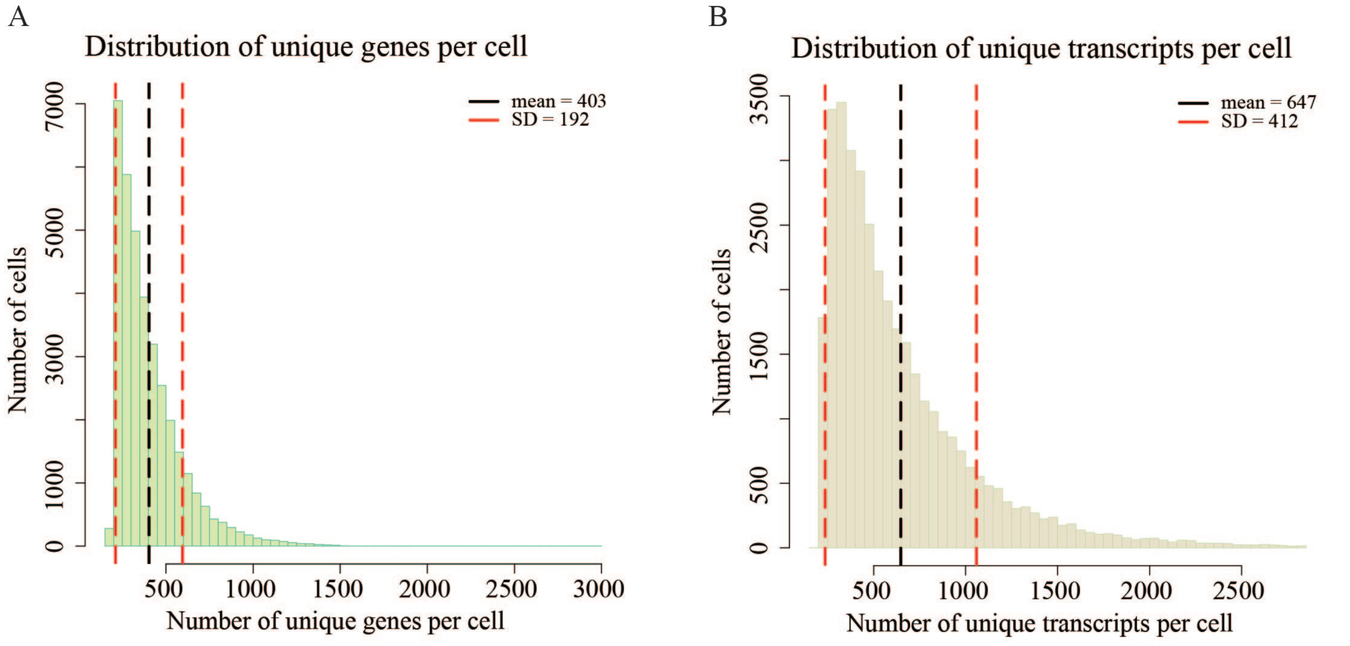

Figure S1. Quality control metrics of GSE225948. **(A)** Distribution of unique genes per cell. **(B)** Distribution of unique transcripts per cell.

The single-cell RNA sequencing (scRNA-seq) dataset GSE154396, derived from a murine stroke model, was assessed for quality control (QC) metrics to evaluate transcriptome complexity and sequencing depth. The dataset exhibits a mean of 1923 unique genes per cell (SD = 623, Figure S2A), indicating a relatively comprehensive gene detection capacity across individual cells. The total unique transcript count per cell averages 4903 UMIs (SD = 2519, Figure S2B), reflecting sufficient sequencing depth for capturing cellular transcriptomes. The observed variability in both gene and transcript counts suggests inherent heterogeneity in transcriptional activity among different cell populations, which is expected in complex tissue microenvironments such as the post-stroke murine brain. These QC results demonstrate that the dataset maintains a high-quality standard, supporting robust downstream analyses, including cell type classification, differential gene expression profiling, and metabolic pathway enrichment studies.

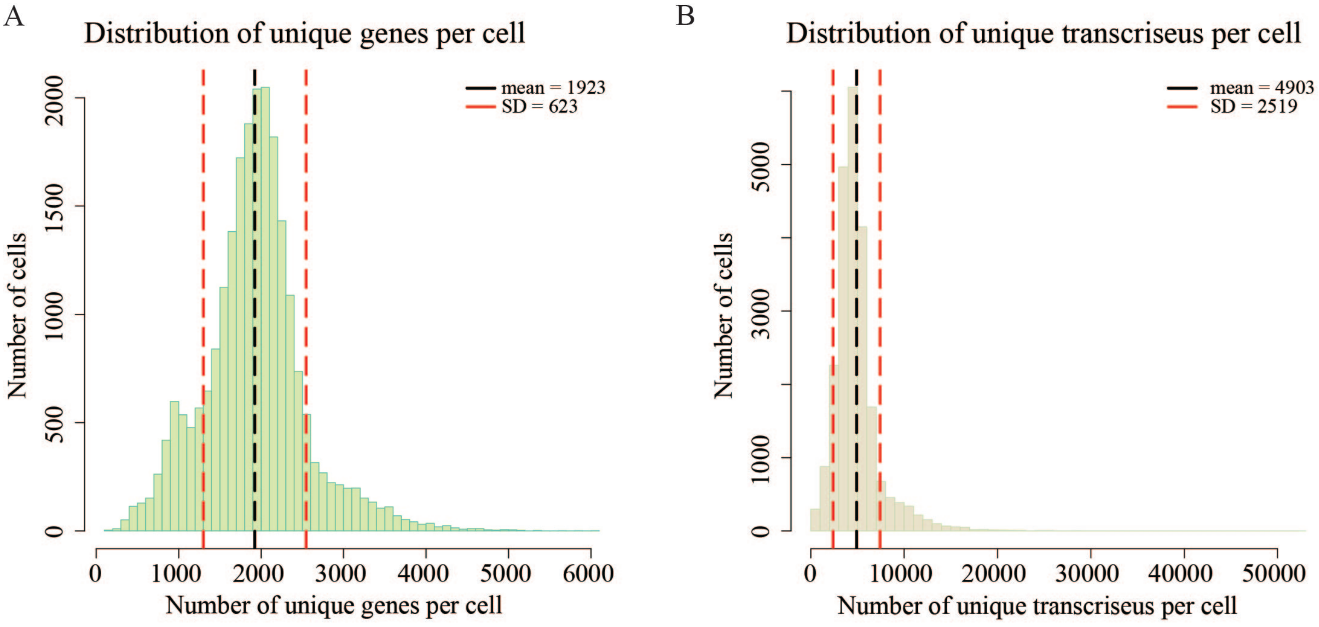

Figure S2. Quality control metrics of GSE154396. **(A)** Distribution of unique genes per cell. **(B)** Distribution of unique transcripts per cell.

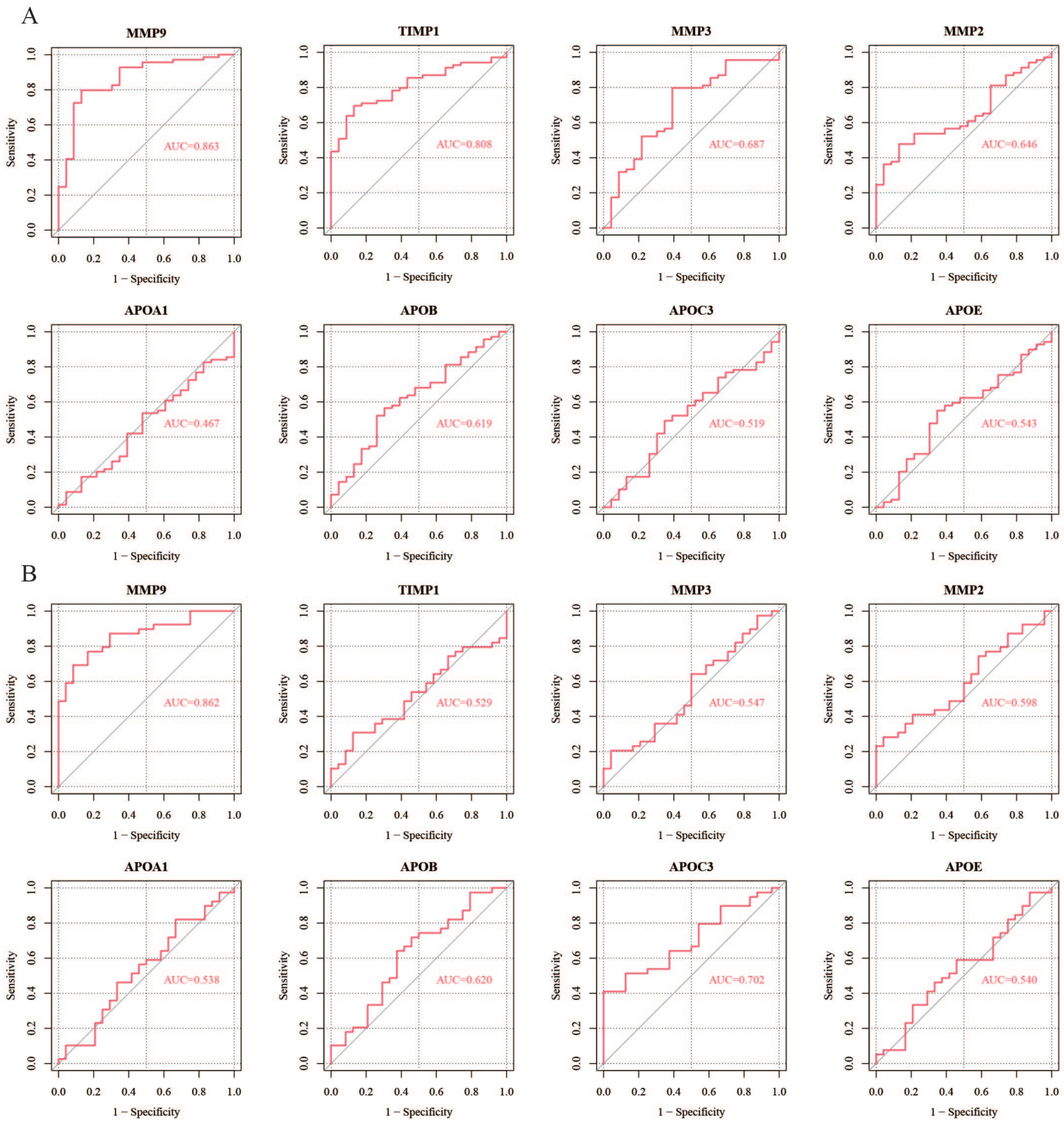

Figure S3. ROC of well-known biomarkers for ischemic stroke. **(A)** ROC of biomarkers in GSE58294. **(B)** ROC of biomarkers in GSE16561.
